# Mitochondria-wide association study observed significant interactions of mitochondrial respiratory and the inflammatory in the development of anxiety and depression

**DOI:** 10.1038/s41398-023-02518-y

**Published:** 2023-06-21

**Authors:** Li Liu, Shiqiang Cheng, Xin Qi, Peilin Meng, Xuena Yang, Chuyu Pan, Yujing Chen, Huijie Zhang, Zhen Zhang, Jingxi Zhang, Chune Li, Yan Wen, Yumeng Jia, Bolun Cheng, Feng Zhang

**Affiliations:** 1 Key Laboratory of Trace Elements and Endemic Diseases, Collaborative Innovation Center of Endemic Disease and Health Promotion for Silk Road Region, School of Public Health, Health Science Center, Xi’an Jiaotong University, Xi’an, 710061 P. R. China; 2 Precision Medicine Center, The First Affiliated Hospital of Xi’an Jiaotong University, Xi’an, P. R. China; 3 Department of Psychiatry, The First Affiliated Hospital of Xi’an Jiaotong University, Xi’an, China

**Keywords:** Human behaviour, Depression

## Abstract

The aim of this study was to investigate the possible interaction of mitochondrial dysfunction and inflammatory cytokines in the risk of anxiety and depression. We utilized the UK Biobank for the sample of this study. A mitochondria-wide association(MiWAS) and interaction analysis was performed to investigate the interaction effects of mitochondrial DNA (mtDNA)×C-reactive protein (CRP) on the risks of self-reported anxiety (*N* = 72,476), general anxiety disorder (GAD-7) scores (*N* = 80,853), self-reported depression (*N* = 80,778), Patient Health Questionnaire (PHQ-9) scores (*N* = 80,520) in total samples, females and males, respectively, adjusting for sex, age, Townsend deprivation index (TDI), education score, alcohol intake, smoking and 10 principal components. In all, 25 mtSNPs and 10 mtSNPs showed significant level of association with self-reported anxiety and GAD-7 score respectively. A total of seven significant mtDNA × CRP interactions were found for anxiety, such as m.3915G>A(*MT-ND1*) for self-reported anxiety in total subjects (*P* = 6.59 × 10^−3^), m.4561T>C(*MT-ND2*) (*P* = 3.04 × 10^−3^) for GAD-7 score in total subjects. For depression, MiWAS identified 17 significant mtSNPs for self-reported depression and 14 significant mtSNPs for PHQ-9 scores. 17 significant mtDNA associations (2 for self-reported depression and 15 for PHQ-9 score) was identified, such as m.14869G>A(*MT-CYB*; *P* = 2.22 × 10^−3^) associated with self-reported depression and m.4561T>C (*MT-ND2*; *P* value = 3.02 × 10^−8^) associated with PHQ-9 score in all subjects. In addition, 5 common mtDNA shared with anxiety and depression were found in MiWAS, and 4 common mtDNA variants were detected to interact with CRP for anxiety and depression, such as m.9899T>C(*MT-CO3*). Our study suggests the important interaction effects of mitochondrial function and CRP on the risks of anxiety and depression.

## Introduction

Anxiety and depression are common neuropsychiatric disorders and global phenomena that often occur simultaneously [1]. Additionally, they are influenced by a combination of multiple genetic and environmental factors that affect certain neural circuits. Poor mental health, such as depression and anxiety disorders, is a significant contributor to the global health-related burden. The Global Burden of Diseases, Injuries, and Risk Factors Study (GBD) 2019 showed depressive and anxiety disorders, two of the most disabling mental disorders, are contributing to the top 10 disease burdens among adolescents [1]. Recent years, much effort has been made to explore the underlying pathologies of anxiety and depression, including inflammation and metabolic change [2], oxidative stress [3], and mitochondrial dysregulation [4]. Despite decades of research, the exact pathophysiology of depression and anxiety is not clearly understood.

Alterations of mtDNA in the bloods and postmortem brain samples have been suggested to be an important factor in the pathogenesis of a range of neuropsychiatric disorders, such as bipolar disorder, depression and anxiety. For example, mitochondrial molecules MFN2 in brain has been shown to regulate anxiety and depression-like behavior through actions on mitochondrial in rat model [5]. A previous study provides data on mtDNA expression and content in occipital cortex samples from major psychiatric disorders patients, and it was found more schizophrenia and major depressive disorder (MDD) patients tended to have the common deletion when compared with control subjects, providing evidence for a role of mtDNA in schizophrenia, bipolar disorder and MDD [6]. The highest mitochondrial DNA deletion burdens were observed in major depressive disorder brain, in addition, brain contained significantly more deletions than blood [7]. Studies showed increased levels of mtDNA content in the hippocampus in patients with bipolar disorder and decreased mtDNA oxidation in the hippocampus in patients with bipolar disorder and schizophrenia [8]. In addition to brain sample, several studies have shown mtDNA alterations in blood cells of patients with anxiety and depression. The mtDNA copy number (mtDNA-cn) was measured using quantitative real-time PCR in peripheral blood in 179 patients with depression, anxiety, and stress- and adjustment disorders and 320 healthy controls, and they found mean mtDNA-cn was significantly higher in patients compared to controls at baseline as well as after controlling for age and sex, indicating the associations between mtDNA-cn and anxiety, depression [9]. Furthermore, mtDNA is released in the extracellular space in different forms, mtDNA may be released at low levels into the circulation from mitochondria under cellular stress as a result of mitochondrial dysfunction or apoptosis, causing elevated circulating cell-free mtDNA (ccf-mtDNA) levels in plasma detectable in MDD [10]. Significantly higher mtDNA copy numbers in blood were seen in individuals with major depression, depressive disorders, and anxiety disorders [11]. Therefore, understanding the various concepts of mitochondrial function in the pathogenesis of mental diseases will undoubtedly help to discover new and more targeted pathogenesis of anxiety and depression. However, despite many efforts, understanding molecular underpinnings of genetic associations on mtDNA is not straightforward. Specifically, it is not entirely clear how mitochondrial function relates to the pathophysiology of anxiety and depression, the extent to which interactions are involved, and whether it has clinical implications.

Growing evidence indicate that elevated concentrations of inflammatory markers are associated with depression and anxiety, including C-reactive protein (CRP) [12], interleukin 6 (IL-6) [13], and other inflammatory markers. CRP is a non-specific acute-phase protein induced by IL-6 in the liver and the active regulator of host innate immunity. Furthermore, the circulating value of CRP reflects ongoing inflammation and/or tissue damage much more accurately than do other laboratory parameters of the acute-phase response [14]. CRP has been widely employed as a biomarker for low-grade inflammation in both psychiatric and physical health conditions [15]. In addition, mendelian randomization analyses suggested that IL-6, CRP are likely to be causally linked with depression [16]. It was found patients with depression and anxiety have higher inflammation than general population [17, 18], measured as proinflammatory cytokines and CRP. Patients of depression showed evidence of low-grade inflammation (>3 mg/L) and mildly elevated CRP levels (>1 mg/L) [19]. Depression is also highly comorbid with anxiety. Nevertheless, for anxiety, associations were mainly limited to CRP and anxiety symptoms of irritability, a symptom also commonly present in depression [2]. The raised CRP levels was mainly found in subjects with generalized anxiety disorder (GAD) [18]. Epidemiology studies also demonstrated the associations between CRP and anxiety [20], depression [2]. Thus, evidence above showed the inflammatory dysregulation contributed to the mechanisms of anxiety and depression.

In the present study, based on the important role of both mitochondrial function and inflammatory in mental disorders, we propose the hypothesis that genetic variations in mtDNA interacted with inflammatory markers (measured by CRP) has important implications for anxiety and depression. To address our hypothesis, we performed the mitochondria-wide association study (MiWAS) of anxiety and depression using mtDNA data, as well as mtDNA×CRP interaction association analysis. Our study may lead to a better understanding of the mitochondrial function in the neuropathophysiological of psychiatric disorders.

## Materials and methods

### UK biobank population

The UK Biobank is a large population-based prospective cohort, constituting ~500,000 individuals aged between 49 and 60 years old in 2007–2010 [21]. Extensive deep genetic and phenotypic data were collected from all participants across the United Kingdom at recruitment. Each participant had a wealth of diverse phenotypic and health-related information, including biometric measurements, lifestyle indicators, biomarkers in blood and urine, and imaging of the body and brain. The signed consents were provided in the participants visit assessment. Ethical approval of UK Biobank was granted by the National Health Service National Research Ethics Service (reference 11/NW/0382). This research has been conducted using the UK Biobank Resource under Application Number 46478. Subjects were excluded if the self-reported gender were inconsistent with the genetic gender, or were genotyped but not imputed or withdraw their consents. Full details of the data collection procedures may refer to the previous study [21].

### UK biobank genotyping, quality control, recalling, and imputation of mtDNA variants

DNA was extracted from buffy coat at UK Biocenter (Stockport, UK) using a Promega Maxwell® 16 Blood DNA Purification Kit (AS1010). UK biobank developed a workflow for quality control (QC), imputation and analysis of mtDNA genotypes that results in a set of variants of similarly high quality to the nuclear-encoded variants for future mtDNA analyses [22]. The workflow uses the standard genotype call, intensity and array manifest files provided after genotyping, as well as publicly available whole-mitochondrial genomes for imputation purposes [22].

Briefly, 265 mtSNVs were genotyped in 488,377 UK biobank participants [23]. A “full set” was generated based existing algorithms [24] to generate a quality-controlled set of 719 mtSNVs in 483,626 pan-ancestry individuals, given the standard procedures for QC and imputation of mtSNVs from genotyping arrays was absent.

For calling and imputation, a four-stage genotype QC procedure was developed: (1) pre-recalling QC, (2) manual recalling, (3) post-recalling QC and (4) imputation of mtSNVs not genotyped on the array. Pre-recalling QC procedures were performed in stage 1 and resulted in 135 poorly called mtSNVs from 265 mtSNVs genotyped in the UK biobank. Then the recalling procedures and post-recalling QC was performed in stage 2 and 3, and 2643 samples with low call rates within each array (call rate ≤0.97) were excluded. Imputation (using IMPUTE2) was performed in stage 4. Detailed information about the genotyping, imputation and quality control of mtDNA variants in UK biobank can be found in a previous published study [22].

### Phenotype definition

Phenotype definition of anxiety (general anxiety disorder (GAD-7) scores; self-reported anxiety), depression (Patient Health Questionnaire (PHQ-9) scores, self-reported depression) were derived from the UK Biobank (https://www.ukbiobank.ac.uk/). GAD-7 and PHQ-9 are effective screening tools for anxiety and depression, respectively, and were used in our study (Detailed information is summarized in the supplementary document).

PHQ-9 and GAD-7 scales are short screening instruments used for detection of depression and anxiety symptoms. Specifically, for depression, PHQ-9 is a rapid and effective tool for screening and monitoring the severity of depression [25]. The PHQ-9 tool is a 9-item screening instrument with a total score (0–27) used to measure the severity of depressive symptoms from no depression to major depressive disorder [26]. Self-reported depression was selected based on the code 1286 from ID 20002, code 3, 4, or 5 from ID 20126 and code 11 from ID 20544 as case. For the control of the self-reported depression, participants meeting criteria for single episode depression and the depression defined in our study were excluded, and participants whose PHQ score ≤5 and did not have core symptoms were selected.

GAD-7 [26] is a classification algorithm with a total score (0–21) used to screen for and measure anxiety severity, focusing on seven anxious symptoms and signs (as detailed below: Feeling nervous, anxious or on edge 20506, Not being able to stop or control worrying 20509, Worrying too much about different things 20520, Trouble relaxing 20515, Being so restless that it is hard to sit still 20516, Becoming easily annoyed or irritable 20505, Feeling afraid as if something awful might happen 20512). Self-reported anxiety was selected based on the code 1287 from ID 20002, and code 15 from ID 20544 as case. For the control group of self-reported anxiety, the generalized anxiety disorder (GAD) and the anxiety defined in our study was excluded, and participants whose GAD score <5 were selected as control group.

### Serum CRP level

Blood concentration of inflammatory marker was measured as high-sensitivity CRP (mg/L) in UK biobank during the baseline data collection. Serum CRP level was measured by immunoturbidimetric high-sensitivity analysis on a Beckman Coulter AU5800. CRP was continuous variable in the present study [27, 28].

### MiWAS and mtDNA × CRP interaction analysis

In the present study, mitochondria-wide association study was conducted using PLINK (https://zzz.bwh.harvard.edu/plink/) [29, 30] female, male and all subjects separately. This method considers mtDNA × CRP interaction effects from a regression model. The genetic additive (ADD) model of PLINK2.0 were used. mtDNAs, CRP and mtDNA × CRP were set as independent variables, anxiety and depression were set as the dependent variables, and sex (not included when calculating interactions in male and female subjects) age, Townsend deprivation index (TDI), education score, alcohol intake, smoke, and principal components were included as covariates. GAD-7 score and PHQ-9 score were used as continuous variables while self-reported anxiety and self-reported depression were used as categorical variables. Thus, linear regression model and logistic regression model were used to estimate the associations between continuable variables, categorical variables and mtDNA × CRP interaction. Formula was set as follows:

$$Y_{{{{\mathrm{ADD}}}}} = \beta _0 + \beta _1 \times {{{\mathrm{mtDNA}}}} + \beta _2 \times {{{\mathrm{CRP}}}} + \beta _3 \times {{{\mathrm{CRP}}}} \times {{{\mathrm{mtDNA}}}}$$

Where *Y* denotes the dependent variables, which are anxiety and depression in the present study. *β*_1 _× mtDNA, *β*_2 _× CRP are the independent factors. *β*_3 _× CRP × mtDNA denotes the interaction effects of two independent variables. Variants with low call rates (<0.90), low Hardy–Weinberg equilibrium exact-test *P* values (<1 × 10^−4^), or low minor-allele frequencies (<0.01) were filtered out. In addition, The unrelated subjects were generated with KING software, a rapid algorithm for relationship inference that allows the presence of unknown population substructure [31]. Significant SNPs were identified at a mitochondria-wide significance threshold of *P* value < 0.05 for MiWAS and *P* value < 0.01 for interaction analysis, given the limited data are available concerning the mtDNA associated with anxiety and depression in UK biobank.

## Results

### Participants

Demographic characteristics of the study sample are displayed in Table 1. Sufficient data in UK biobank were available for 72,476 participants for self-reported anxiety, 80,853 participants for GAD score, 80,778 participants for self-reported depression, and 80,520 participants for PHQ-9 score, respectively.

**Table 1:** Demographic characteristics of the population of UK biobank in this study.

Phenotype	Anxiety		Depression	
	Self-reported anxiety (case:contorl)	GAD scores	Self-reported depression (case:contorl)	PHQ-9 scores
*N*	72,476 (13,610:58,866)	80,853	80,778 (36,993:43,785)	80,520
Sex (female/male)	38,478/33,998	43,679/37,174	44,086/36,692	43,505/37,015
Age (years; mean ± SD)	56.468 ± 7.540	56.212 ± 7.585	56.432 ± 7.619	56.219 ± 7.585
CRP (mg/L; mean ± SD)	2.204 ± 3.883	2.201 ± 3.881	2.347 ± 4.028	2.197 ± 3.873

*CRP* C-reactive protein, *GAD score* general anxiety disorder score, *PHQ-9 score* Patient Health Questionnaire scores.

### MiWAS results for anxiety

**Fig. 1:**
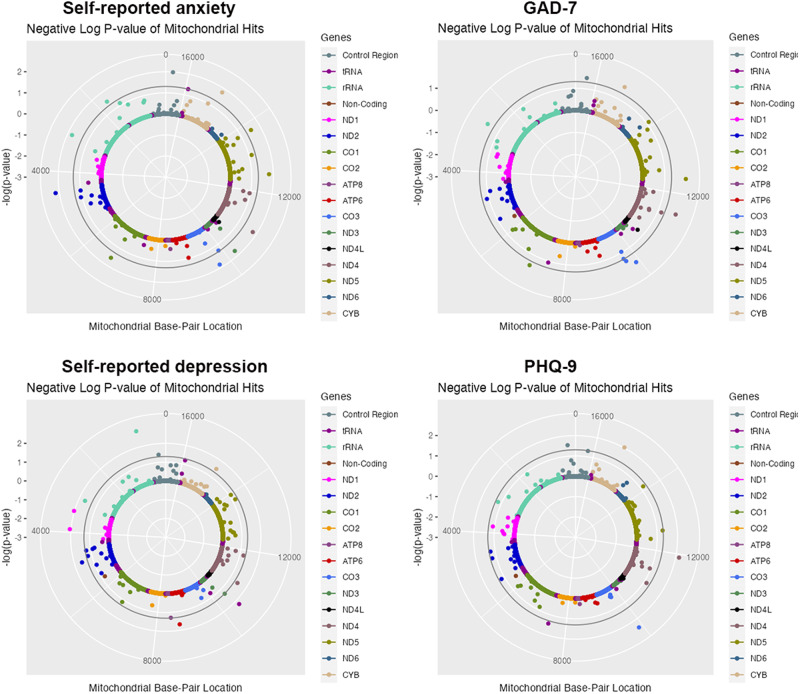
Solar Manhattan plot of the association *p*-values between mtDNA variants and depression, anxiety in total subjects. Each dot represents a mtDNA variant association with anxiety or depression, color-coded by gene. The basepair positions are provided on a circular x-axis. The −log10(p)-values are provided on a radial *y*-axis, which is the distance from the center. The significant threshold was set as *p* < 0.05, and is represented by the circular gray line.

25 mtSNPs and 10 mtSNPs showed significant level of association with self-reported anxiety and GAD score separately (Tables 2 and S1 and Fig. 1). For example, m.16391G>A(*MT-DLOOP*) was found to be associated with self-reported anxiety in total subjects (*P* value = 1.03 × 10^−2^) and female subjects (*P* value = 7.69 × 10^−3^). m.16270 C > T(*MT-DLOOP*) was found to be associated with GAD score in total subjects (*P* value = 3.24 × 10^−2^) and female subjects (*P* value = 2.08 × 10^−2^).

**Table 2:** Top 3 mtDNAs identified in MiWAS for anxiety (*p* < 0.05).

Gender	Phenotype	Variant	mtDNA	BETA	SE	*P* value
Total	Self-reported anxiety	m.4529A>T	*MT-ND2*	−0.02744	0.01011	6.66 × 10^−3^
Total	Self-reported anxiety	m.16391G>A	*MT-DLOOP*	−0.02562	0.009986	1.03 × 10^−2^
Total	Self-reported anxiety	m.9716T>C	*MT-CO3*	0.03354	0.0137	1.43 × 10^−2^
male	Self-reported anxiety	m.5999T>C	*MT-CO1*	−0.03506	0.01652	3.38 × 10^−2^
male	Self-reported anxiety	m.14620C>T	*MT-ND6*	−0.03436	0.01636	3.58 × 10^−2^
male	Self-reported anxiety	m.15693T>C	*MT-CYB*	−0.03458	0.01652	3.64 × 10^−2^
female	Self-reported anxiety	m.15218A>G	*MT-CYB*	0.03779	0.01308	3.88 × 10^−3^
female	Self-reported anxiety	m.16391G>A	*MT-DLOOP*	−0.03928	0.01474	7.69 × 10^−3^
female	Self-reported anxiety	m.4529A>T	*MT-ND2*	−0.03911	0.01496	8.96 × 10^−3^
Total	GAD-7	m.12372G>A	*MT-ND5*	0.08378	0.03315	1.15 × 10^−2^
Total	GAD-7	m.11467A>G	*MT-ND4*	0.08013	0.03327	1.60 × 10^−2^
Total	GAD-7	m.9899T>C	*MT-CO3*	−0.2587	0.1105	1.92 × 10^−2^
female	GAD-7	m.4580G>A	*MT-ND2*	0.2637	0.1101	1.66 × 10^−2^
female	GAD-7	m.16270C>T	*MT-DLOOP*	0.1629	0.07044	2.08 × 10^−2^
female	GAD-7	m.9716T>C	*MT-CO3*	0.3317	0.1564	3.39 × 10^−2^

GAD-7 means general anxiety disorder score.

### mtDNA×CRP interaction analysis results for anxiety

**Fig. 2:**
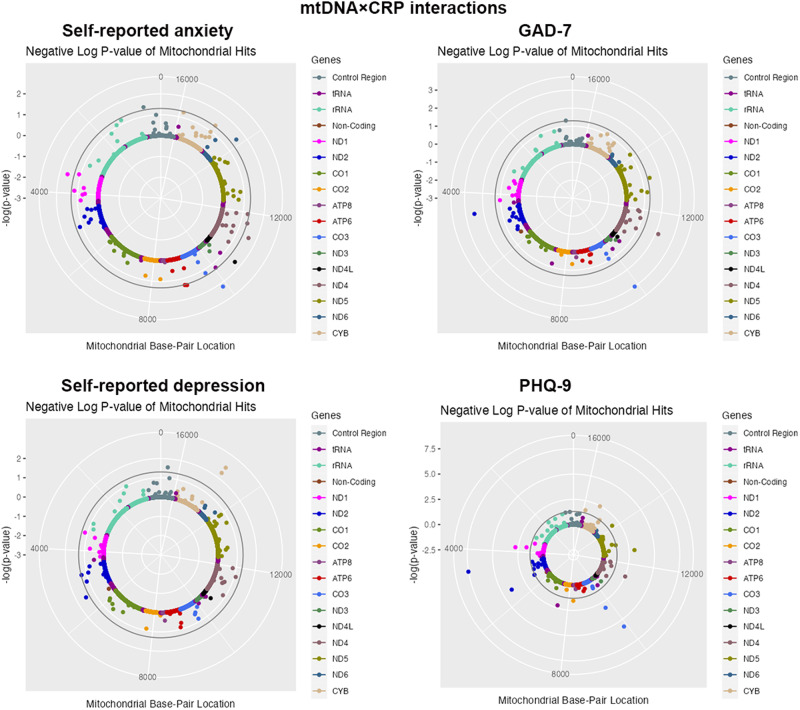
Solar Manhattan plot of the association p-values between mtDNA×CRP interactions and anxiety, depression in total subjects. Each dot represents a mtDNA variant association with anxiety or depression, color-coded by gene. The basepair positions are provided on a circular *x*-axis. The −log10(*p*)-values are provided on a radial *y*-axis, which is the distance from the center. The significant threshold was set as *p* < 0.05, and is represented by the circular gray line.

A total of 7 mtDNA–CRP associations were detected for anxiety (Table 3) (Fig. 2), such as m.3915G>A(*MT-ND1*) with self-reported anxiety in all subjects (*P* value = 6.59 × 10^−3^), m.12633C>A (*MT-ND5*; *P* value = 4.90 × 10^−3^), and m.11467A>G (*MT-ND4*; *P* value = 9.39 × 10^−3^) with self-reported anxiety in male participants. 3 mtDNA–CRP associations were found for GAD score in all subjects, such as m.4561T>C(*MT-ND2*; *P* value = 3.04 × 10^−3^), m.11377G>A(*MT-ND4*; *P* value = 6.93 × 10^−3^).

**Table 3:** Results of mtDNA×CRP interaction analysis for anxiety in UK biobank (*p* < 0.01).

Gender	Phenotype	Variant	mtDNA	BETA	SE	*P* value
Total	Self-reported anxiety	m.3915G>A	*MT-ND1*	0.0104	0.0038	6.59 × 10^−3^
Male	Self-reported anxiety	m.12633C>A	*MT-ND5*	0.0167	0.0059	4.90 × 10^−3^
Male	Self-reported anxiety	m.9899T>C	*MT-CO3*	0.0161	0.0059	6.00 × 10^−3^
Male	Self-reported anxiety	m.11467A>G	*MT-ND4*	0.0128	0.0049	9.39 × 10^−3^
Total	GAD-7	m.9899T>C	*MT-CO3*	0.0994	0.0302	9.95 × 10^−4^
Total	GAD-7	m.4561T>C	*MT-ND2*	0.0759	0.0256	3.04 × 10^−3^
Total	GAD-7	m.11377G>A	*MT-ND4*	−0.0564	0.0209	6.93 × 10^−3^

CRP means C-reactive protein. GAD-7 means general anxiety disorder score.

### MiWAS results for depression

17 mtSNPs and 14 mtSNPs showed significant level of association with self-reported depression and PHQ score separately (Tables 4 and S2 and Fig. 1). m.709G>A(*MT-RNR1*) was associated with self-reported depression in total subjects (*P* value = 1.40 × 10^−3^), male (*P* value = 8.77 × 10^−3^) and female subjects (*P* value = 4.48 × 10^−2^). m.9899T>C(*MT-CO3*) was also detected to be associated with PHQ-9 score in total subjects with a *p* value of 3.94 × 10^−3^ and in female subjects with a *p* value of 2.81 × 10^−2^.

**Table 4:** Top3 mtDNAs identified in MiWAS for depression (*p* < 0.05).

Gender	Phenotype	Variant	mtDNA	BETA	SE	*P* value
Total	Self-reported depression	m.709G>A	*MT-RNR1*	0.0151	0.004715	1.40 × 10^−3^
Total	Self-reported depression	m.10463T>C	*MT-TR*	0.015	0.005443	5.79 × 10^−3^
Total	Self-reported depression	m.3915G>A	*MT-ND1*	0.0264	0.01005	8.74 × 10^−3^
male	Self-reported depression	m.3915G>A	*MT-ND1*	0.0403	0.01489	6.86 × 10^−3^
male	Self-reported depression	m.709G>A	*MT-RNR1*	0.0182	0.00695	8.77 × 10^−3^
male	Self-reported depression	m.13965T>C	*MT-ND5*	0.0428	0.02182	4.97 × 10^−2^
female	Self-reported depression	m.3394T>C	*MT-ND1*	−0.043	0.01967	2.73 × 10^−2^
female	Self-reported depression	m.10463T>C	*MT-TR*	0.0156	0.007394	3.44 × 10^−2^
female	Self-reported depression	m.3010G>A	*MT-RNR2*	−0.011	0.00518	3.85 × 10^−2^
Total	PHQ-9	m.9899T>C	*MT-CO3*	−0.336	0.1166	3.94 × 10^−3^
Total	PHQ-9	m.11914G>A	*MT-ND4*	0.2986	0.1103	6.81 × 10^−3^
Total	PHQ-9	m.15257G>A	*MT-CYB*	0.2538	0.0978	9.47 × 10^−3^
male	PHQ-9	m.11914G>A	*MT-ND4*	0.5641	0.1508	1.84 × 10^−4^
male	PHQ-9	m.6776T>C	*MT-CO1*	−0.221	0.09361	1.85 × 10^−2^
male	PHQ-9	m.73A>G	*MT-DLOOP*	−0.097	0.04183	2.06 × 10^−2^
female	PHQ-9	m.4580G>A	*MT-ND2*	0.3174	0.1124	4.74 × 10^−3^
female	PHQ-9	m.16193C>T	*MT-DLOOP*	0.4135	0.1805	2.20 × 10^−2^
female	PHQ-9	m.9899T>C	*MT-CO3*	−0.377	0.1716	2.81 × 10^−2^

PHQ-9 score means Patient Health Questionnaire scores.

### mtDNA×CRP interaction analysis results for depression

A total of 17 mtDNA associations was identified for depression (Table 5 and Fig. 2), including 2 mtDNA associations for self-reported depression and 15 mtDNA associations for PHQ-9 score. m.14869G>A(*MT-CYB*) was found in both all subjects (*P* value = 2.22 × 10^−3^) and female subjects (*P* value = 5.57 × 10^−3^) with self-reported depression. m.4561T>C (*MT-ND2*) was detected in both all subjects (*P* value = 3.02 × 10^−8^) and male subjects (*P* value = 3.25 × 10^−7^) with PHQ-9 score.

**Table 5:** Results of mtDNA×CRP interaction analysis for depression in UK biobank (*p* < 0.01).

Gender	Phenotype	Variant	mtDNA	BETA	SE	*P* value
Total	Self-reported depression	m.14869G>A	*MT-CYB*	−0.0112	0.0037	2.22 × 10^−3^
Female	Self-reported depression	m.14869G>A	*MT-CYB*	−0.0130	0.0047	5.57 × 10^−3^
Total	PHQ-9	m.4561T>C	*MT-ND2*	0.1462	0.0264	3.02 × 10^−8^
Total	PHQ-9	m.9899T>C	*MT-CO3*	0.1508	0.0318	2.15 × 10^−6^
Total	PHQ-9	m.12633C>A	*MT-ND5*	0.1120	0.0338	9.17 × 10^−4^
Total	PHQ-9	m.3796A>G	*MT-ND1*	0.0803	0.0255	1.60 × 10^−3^
Total	PHQ-9	m.11377G>A	*MT-ND4*	−0.0634	0.0213	2.94 × 10^−3^
Total	PHQ-9	m.15257G>A	*MT-CYB*	−0.0536	0.0183	3.40 × 10^−3^
Total	PHQ-9	m.7476C>T	*MT-TS1*	−0.0576	0.0209	5.76 × 10^−3^
Male	PHQ-9	m.4561T>C	*MT-ND2*	0.1724	0.0337	3.25 × 10^−7^
Male	PHQ-9	m.9899T>C	*MT-CO3*	0.1458	0.0392	2.03 × 10^−4^
Female	PHQ-9	m.5495T>C	*MT-ND2*	0.1479	0.0392	1.62 × 10^−4^
Female	PHQ-9	m.3796A>G	*MT-ND1*	0.1166	0.0311	1.74 × 10^−4^
Female	PHQ-9	m.12633C>A	*MT-ND5*	0.1930	0.0521	2.11 × 10^−4^
Female	PHQ-9	m.16356T>C	*MT-DLOOP*	0.0713	0.0226	1.60 × 10^−3^
Female	PHQ-9	m.9899T>C	*MT-CO3*	0.1663	0.05272	1.61 × 10^−3^
Female	PHQ-9	m.15924A>G	*MT-TT*	0.0578	0.0210	5.97 × 10^−3^
Female	PHQ-9	m.11377G>A	*MT-ND4*	−0.0734	0.0278	8.22 × 10^−3^

CRP means C-reactive protein. PHQ-9 score means Patient Health Questionnaire scores.

### Common mtDNA variants shared with anxiety and depression

**Fig. 3:**
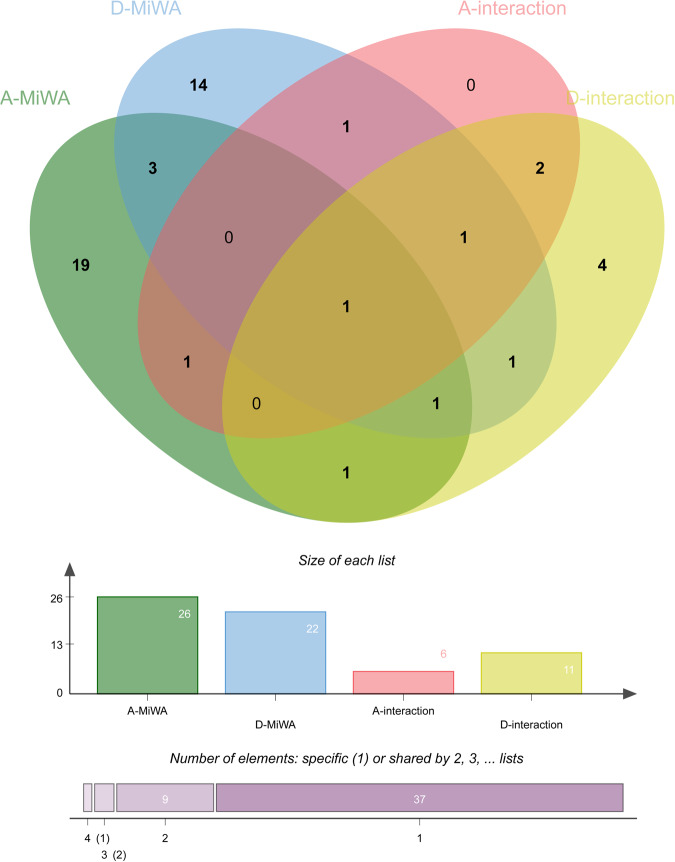
Venn diagrams of genes shared by the MiWAs analysis and mtDNA×CRP interaction analysis in anxiety and depression. Venn diagrams of genes shared by the above four modules are the differentially expressed mtDNAs for anxiety (A-MiWAS) and depression (D-MiWAS) in MiWAS, respectively. Differentially expressed mtDNAs interacted with CRP for anxiety in interaction analysis (A-interaction) and for depression in interaction analysis (D-interaction).

There were 5 common mtDNAs shared with anxiety and depression in MiWAS (Fig. 3), namely m.10915T>C(*MT-ND4*), m.3010G>A(*MT-RNR2*), m.9899T>C(*MT-CO3*), m.15257G>A(*MT-CYB*) and m.4580G>A(*MT-ND2*). In addition, 4 mtDNA variants were detected to interact with CRP for both anxiety and depression, including m.12633C>A(*MT-ND5*), m.9899T>C(*MT-CO3*), m.4561T>C(*MT-ND2*), m.11377G>A(*MT-ND4*). For example, m.12633C>A (*MT-ND5*) was identified to interact with CRP for both in male subjects with self-reported anxiety (*P* value = 4.90 × 10^−3^) and all subjects with PHQ-9 score (*P* value = 9.17 × 10^−4^). m.9899T>C (*MT-CO3*) was identified as a common mtDNA variant interacted with CRP in male subjects with self-reported anxiety (*P* value = 6.00 × 10^−3^), all subjects with GAD score(*P* value = 9.95 × 10^−4^), and all subjects with PHQ-9 score (*P* value = 2.15 × 10^−6^), male subjects with PHQ-9 score (*P* value = 2.03 × 10^−4^), female subjects with PHQ-9 score (*P* value = 1.61 × 10^−3^). m.4561T>C (*MT-ND2*) was detected in both all subjects (*P* value = 3.02 × 10^−8^), male subjects (*P* value = 3.25 × 10^−7^) with PHQ-9 score, and all subjects with GAD score (*P* value = 3.04 × 10^−3^). m.11377G>A (*MT-ND4*) was found in both all subjects with GAD score (*P* value = 6.93 × 10^−3^), total subjects (*P* value = 2.94 × 10^−3^) and female subjects with PHQ-9 score (*P* value = 8.22 × 10^−3^).

## Discussion

In the present study, we found several significant mtDNA × CRP interactions for anxiety and depression respectively. Our findings have important implications for explaining the involvement of inflammatory cytokines and mitochondrial respiratory chain complex in the pathophysiological mechanisms of anxiety and depression.

There is growing recognition that mitochondrial dysfunction and neuroinflammation do not act alone and may be intricately interacts with each other. Observational and experimental studies indicated the associations between mitochondrial respiratory and inflammatory cytokines in mental disorders. Scaini et al. [32] found MDD patients with higher CRP levels had higher levels of mitochondrial molecules *Mfn-2* and *LC3B* when compared with MDD patients with low CRP, indicating the alterations in the mitochondrial fragmentation due to CRP level in MDD patients. Furthermore, they observed the severity of depressive symptoms in MDD is also linked with changes in protein levels in pathways related to mitochondrial dynamics and mitophagy, and may dependent on inflammatory status [32]. The impaired mitochondrial functioning was found in peripheral blood mononuclear cells (PBMCs) of patients with MDD, including lower routine and uncoupled respiration, spare respiratory capacity, coupling efficiency, and adenosine triphosphate (ATP) turnover-related respiration. In addition, mitochondrial respiration correlated negatively with depressive symptom severity, indicating the essential role of mitochondrial respiration in the blunted immunity in depression [33]. Furthermore, another study revealed the impaired mitochondrial respiration in thrombocytes of depressed individuals, which might be participating in pathophysiology of depression [34]. Consequently, an increasing body of literature implicates inflammatory-mitochondrial dysfunction interactions as an important contributing factor to the underlying mechanisms of psychopathologies. Our study results suggested the contribution of mtDNA × CRP interactions in the development of anxiety and depression. However, direct evidence concerning the interactions between CRP and mitochondrial respiratory are not reported in previous studies. Experimental studies are required to further evaluate causality, mechanisms underlying this interaction.

Possible mechanisms underlying the interactions between CRP and mtDNA for mental disorders are not fully clarified. Several hypotheses have been proposed. First, mtDNA regulates energy production and cell metabolism and can directly modulate immune response. Specifically, mitochondria possess bacterial characteristics, and mitochondrial-derived damage-associated molecular patterns (DAMPs) are recognized by immune receptors of microglia and resulting in the expression of inflammatory mediators, thus amplifying neuroinflammation [35]. In addition, experiment studies showed mitochondrial dysfunction leads to increased pro-inflammatory activity [36]. Second, inflammation affects mitochondrial energy metabolism [37]. The continuous production of toxic mediators such as reactive oxygen species (ROS) and cytokines inhibit and destroys mitochondria [38, 39]. A growing body of evidence indicates that inflammation may further cause deleterious changes in mitochondrial function, affecting oxidative phosphorylation and membrane polarity [37]. Moreover, inflammatory regulates the release of mtDNA from mitochondria [40] and the release of mitochondrial proteins into the cytoplasm and/or extracellular environment is a significant activator of inflammation [41]. Chen et al. found monomeric CRP can bind platelets via interaction with lipid raft and induce the release of mtDNA [42]. Thus, evidence above demonstrated the crosstalk between neuroinflammatory and mtDNA. However, specific mechanisms underlying the interactions is unclear. Further additional experiments and analyses are needed to explore the potential mechanisms underlying the interaction between mtDNA and CRP, and to validate the findings of previous studies.

We further identified several significant mtDNA×CRP associations for anxiety and depression, including mitochondrial gene *MT-ND2* for PHQ-9 and *MT-CYB* for self-reported depression, *MT-ND1* for self-reported anxiety, *MT-CO3* for GAD score. In most organisms, the mitochondrial respiratory chain (MRC) is composed of four complexes (I, II, III, and IV), namely MRC complex I (*MT-ND1*, *MT-ND5*, and *MT-ND6*), complex II (succinate dehydrogenase (SDH)), complex III (*MT-CYB*), and complex IV (CIV) subunits (*MT-CO2* and *MT-CO3*), where the electron transport couples with translocation of protons from the mitochondrial matrix to the intermembrane space. Our study results indicate the important role of mitochondrial respiratory chain (MRC) int the interaction association. For example, *MT-CYB*, which was found to interact with CRP for self-reported depression, is the only mitochondrial DNA encoded subunit of respiratory complex III. In addition, *MT-CO3* is part of respiratory chain complex IV, was found to interact with CRP for GAD score and PHQ-9. Increasing evidence suggests damage to the mitochondrial electron transport chain is an important factor in the pathogenesis of a range of psychiatric disorders. The relationship between MRC, inflammatory and mood disorders has been studies before. A recent study have linked mitochondrial electron transport chain function to NLRP3 inflammasome activation [43], they found mitochondrial complex I function is required for caspase-1 activation and production of secreted IL-1β protein [43]. Another study compared the MRC activities, mtDNAcn, and the composite the Mitochondrial Health Index (MHI) in subjects with MDD and healthy controls as well as SSRI antidepressant responders and non-responders, and they identified significantly higher baseline mitochondrial content markers citrate synthase (CS) and mtDNAcn, and higher complex I activity in SSRI responders when compared to SSRI non-responders [44]. Our study confirmed the important role of MRC in the development of anxiety and depression.

*MT-ND1* and *MT-ND2* encode two subunits of NADH dehydrogenase play an important role in the electron transport chain of oxidative phosphorylation (OXPHOS). Mitochondria are key providers of energy to the cell in the form of ATP through OXPHOS. Patients with age-related neurodegenerative diseases, such as Parkinson’s disease (PD) and Alzheimer’s disease (AD), have reduced expression of the gene for the mtDNA OXPHOS protein in their brain tissues [45]. And OXPHOS dysfunction can produce the ROS and oxidative stress, leading to neuronal cell death [46]. Previous study showed imbalance in nuclear and mitochondrial genome-encoded OXPHOS transcripts may reduce mitochondrial translation and compromise OXPHOS efficiency, which is likely to generate damaging reactive oxygen species [47]. Another study indicates increased expressions in certain mt genes and elevated levels of ROS may potentially play a critical role in AD progression [48]. In addition to mt genes, the imbalance in ROS also leads to abnormality of brain functions and neuronal signaling [49], thus implicating in the progression of psychiatric diseases [49, 50]. Generation of ROS is a necessary biological process. Furthermore, ROS production have been suggested as a contributing factor in immunogenic cell death and T cell-mediated immunity [51]. Since the important role of ROS in depression and anxiety, as well as the associations between ROS and inflammatory, based on these phenomena, it is plausible that the interaction between *MT-ND1* and *MT-ND2* and CRP may occur by acting on ROS for mood disorders. As is often the case, the answer may lie in a combination of these possibilities.

It is well-known that anxiety and depression is more prevalent in female subjects. Thus, it is necessary to further perform sex stratified analyses. In fact, accumulated studies demonstrated the sex-dependent in mitochondrial function or dysfunction, which may determine sex differences in psychiatric pathologies [52]. For example, the sex-related difference of mitochondrial DNA copy number(mtDNAcn) was examined in post-traumatic stress disorder (PTSD) patients, with significantly higher mtDNAcn in female subjects with PTSD compared to male or female non-PTSD controls or male subjects with PTSD [53]. However, rare previous studies have ever investigated potential sex differences in mtDNA under anxiety-related or depression-related psychiatric illness. A previous study from our group confirmed the potential sex-related differences in the associations between mitochondrial function and human behavior interactions and anxiety/depression [54]. We found mtDNA × CRP interactions differed in male, female and total subjects with anxiety or depression. For example, a total of 7, 2, 7 mtDNA × CRP interactions were found in total subjects, male subjects and female subjects with PHQ-9 respectively in the present study. Among them, m.9899T>C(*MT-CO3*) was the common mtDNA signature spanning all three groups. Further research may investigate whether the mtDNA × CRP interactions provide a mechanistic explanation to the sex difference in the prevalence of anxiety and depression.

This study is one of the first to systematically explore the associations among inflammatory, CRP and mood disorders using mitochondria-wide association study and mtDNA×CRP association analysis. Many studies have investigated the association between serum CRP level and psychiatric disorders, but few focused on the role of mt DNA. It is important to note that we examined here a new aspect of pathogenesis of mood disorders-mitochondrial functioning × inflammatory interaction associations. The interaction between mtDNA and CRP is discussed as a promising new biomarker for diagnosis and treatment effects in anxiety and depression. Our study will enhance future studies into the role of inflammatory and mitochondrial function in the development of mood disorders.

While promising, there are still a few issues need to be attention. First, the number of study subjects used in this study is relatively small owing to the limited mtDNA subjects in the UK biobank. In addition, we restricted our subjects to white European ancestry population, which should not be generalized to other ethnics. Second, we did not have specific experiment results regarding mtDNA × CRP associations for mood disorders, in addition, the observed mtDNA × CRP interactions in the presents study do not necessarily mean causality. So, these findings should be considered preliminary and in need of replication. Further studies are required to elucidate these possible and biologically plausible associations.

## Conclusions

Overall, we performed the MiWAS and mtDNA × CRP interaction association analysis using mtDNA data. Several significant mtDNA × CRP associations were found for anxiety and depression, respectively. Our study confirmed the interaction role of mitochondrial function and inflammatory cytokines (CRP) in the neuropathophysiological of psychiatric disorders.

## Supplementary information

Phenotypes definition of GAD-7 scores and PHQ-9 scores in UK biobank.

Supplementary tables legends

Supplementary tables

## Data Availability

The UKB data are available through the UK Biobank Access Management System (https://www.ukbiobank.ac.uk/). We will return the derived data fields following UKB policy; in due course, they will be available through the UK Biobank Access Management System. The datasets used and/or analyzed during the current study are available from the corresponding author on reasonable request.
