# Supplementary material for: Mitochondria-wide association study observed significant interactions of mitochondrial respiratory and the inflammatory in the development of anxiety and depression
Source: Transl Psychiatry. 2023 Jun 21;13:216. doi: 10.1038/s41398-023-02518-y (PMC10284875; doi:10.1038/s41398-023-02518-y)
Supplement: Supplementary file 1 — Phenotypes definition of GAD-7 scores and PHQ-9 scores in UK biobank. [file 41398_2023_2518_MOESM1_ESM.docx]

# Appendix:

Section A: anxiety (GAD-7)

Section B: depression (PHQ-9)

| **Generalized anxiety disorder-7(GAD-7).** | | | | |
| --- | --- | --- | --- | --- |
| 1. **20506** 2. **20509** 3. **20520** 4. **20515** 5. **20516** 6. **20505** 7. **20512** | Over the last 2 weeks, how often have you been bothered by any of the following problems?  a) Feeling nervous, anxious or on edge  b) Not being able to stop or control worrying  c) Worrying too much about different things  d) Trouble relaxing  e) Being so restless that it is hard to sit still  f) Becoming easily annoyed or irritable  g) Feeling afraid as if something awful might happen  [7 questions on one screen in grid] | | | [Select one from the following for each of the statements]  - 01 Not at all  - 02 Several days  - 03 More than half the days  - 04 Nearly every day  - DA Prefer not to answer |
| **Patient health questionnaire-9(PHQ-9):** | | | | |
| 1. **20514** 2. **20510** 3. **20534** 4. **20519** 5. **20511** 6. **20507** 7. **20508** 8. **20518** 9. **20513** | | Over the last 2 weeks, how often have you been bothered by any of the following problems?  a. Little interest or pleasure in doing things  b. Feeling down, depressed, or hopeless  c. Trouble falling or staying asleep, or  sleeping too much  d. Feeling tired or having little energy  e. Poor appetite or overeating  f. Feeling bad about yourself or that you  are a failure or have let yourself or your  family down  g. Trouble concentrating on things, such  as reading the newspaper or watching  television  h. Moving or speaking so slowly that other  people could have noticed? Or the opposite — being so fidgety or restless that you have been moving around a lot more than usual  i. Thoughts that you would be better off  dead or of hurting yourself in some way | [Select one from the following for each of the statements]  - 01 Not at all  - 02 Several days  - 03 More than half the days  - 04 Nearly every day  - DA Prefer not to answer | |
