# Supplementary material for: Mitochondria-wide association study observed significant interactions of mitochondrial respiratory and the inflammatory in the development of anxiety and depression
Source: Transl Psychiatry. 2023 Jun 21;13:216. doi: 10.1038/s41398-023-02518-y (PMC10284875; doi:10.1038/s41398-023-02518-y)
Supplement: Supplementary file 2 — Supplementary tables legends [file 41398_2023_2518_MOESM2_ESM.docx]

**Supplementary tables**

**Table S1:** MiWAS results for anxiety (p<0.05)

**Table S2:** MiWAS results for depression (p<0.05)

**Supplementary materials:** Phenotypes definition of GAD-7 scores and PHQ-9 scores in UK biobank.
